# Self-Evolving Multi-Agent Fuzzing for Industrial IoT with Knowledge-Driven Cognitive Reasoning

**DOI:** 10.3390/s26113348

**Published:** 2026-05-25

**Authors:** Bowei Ning, Xuejun Zong, Kan He, Guogang Wang, Lian Lian, Yifei Sun, Jinyang Liu

**Affiliations:** 1 School of Artificial Intelligence, Shenyang University of Technology, Shenyang 110870, China; 2020183@stu.syuct.edu.cn (B.N.);; 2 Key Laboratory of Information Security for Petrochemical Industry in Liaoning Province, Shenyang 110142, China; 3 School of Information Engineering, Shenyang University of Chemical Technology, Shenyang 110142, China

**Keywords:** Industrial Internet of Things (IIoT) security, multi-agent systems, protocol fuzzing, large language models, knowledge graph, trustworthy AI, vulnerability discovery

## Abstract

Securing the Industrial Internet of Things (IIoT) is paramount, yet proprietary protocols remain vulnerable to deep-state logic flaws that traditional fuzzers often fail to reach. We propose MALF, a Multi-Agent LLM Fuzzing Framework that couples a dynamic Industrial Security Knowledge Graph (ISKG) with collaborative cognitive agents for effective, efficient, and trustworthy IIoT security testing. A self-evolving knowledge loop mitigates LLM hallucinations by grounding the generation in verifiable graph constraints; QLoRA-tuned models aligned with hexadecimal features enable low-latency mutation; and Chain-of-Thought reasoning reconstructs protocol states for intent-driven attacks. On a heterogeneous testbed spanning five industrial protocols and ten vendors, MALF achieves an average Test Case Acceptance Rate of 88.3% (peak 91.2% on Modbus/TCP) and 91.2% ISKG-defined state coverage, outperforming rule-based, RL-based, and LLM baselines. On a 15-vulnerability N-Day benchmark, MALF detects all known cases, against 60%, 47%, 40%, and 27% for NCMFuzzer, MARLFuzz, BooFuzz, and Fuzz4All, respectively. In a separate real-world campaign, MALF further identifies 14 previously unknown vulnerability candidates, of which four have been assigned CNVD identifiers (CNVD-2024-16009, CNVD-2025-22875, CNVD-2025-29811, CNVD-2026-06041) and 10 remain under vendor review. These results provide controlled-testbed evidence that knowledge-grounded AI agents can systematically expose deep-state vulnerabilities in opaque IIoT environments.

## 1. Introduction

The architecture of Industrial Control Systems (ICS) is undergoing a fundamental transformation from the traditional hierarchical ISA-95 model to dynamic, networked Industrial Internet of Things (IIoT) frameworks. While this transformation enhances operational efficiency and real-time interconnection [1], it simultaneously expands the attack surface—the set of network endpoints, protocol parsers, and exposed services through which an adversary can interact with critical infrastructure—and thereby enlarges the space of exploitable entry points. Key Industrial Control Protocols (ICPs), serving as the communication backbone of this ecosystem, enable cross-layer interoperability but rely on deterministic, stateful communication patterns. These protocols introduce unique security risks; for instance, the lack of native encryption in Modbus/TCP and the complex metadata structures in OPC UA create vulnerabilities that can be exploited to disrupt safety-critical processes [2,3]. Consequently, ensuring the security of these connected devices is paramount for the reliability of the IIoT ecosystem.

Currently, fuzz testing serves as a pivotal dynamic technique for exposing such weaknesses [4]. However, applying traditional fuzzing to the heterogeneous IIoT environment presents distinct challenges regarding effectiveness and efficiency [5]. Unlike standard IT protocols, ICPs require strict adherence to message sequences, timing constraints, and context-dependent structures [6]. Traditional mutation-based fuzzers like Peach [7] rely on rigid, heuristic rules that often fail to generate semantically valid seeds for highly structured headers. This limitation results in inefficient mutation processes and shallow code coverage, leaving deep-state logic vulnerabilities undetected [8].

The integration of large language models (LLMs) offers a promising avenue to surmount these obstacles [9]. Nevertheless, significant gaps remain. Off-the-shelf LLMs lack domain knowledge of proprietary protocols, often leading to “hallucinations”—syntactically correct but semantically invalid packets—which undermines the trustworthiness of the testing process [10]. While Retrieval-Augmented Generation (RAG) has been proposed to mitigate this, traditional text-based retrieval struggles to capture multi-hop dependencies inherent in industrial state machines. Furthermore, existing single-agent approaches lack the collaborative architecture required for efficient decision-making [11].

To address these limitations, this work presents MALF, a novel framework leveraging multi-agent LLMs for effective, efficient, and trustworthy fuzz testing of ICPs. Unlike monolithic approaches, MALF utilizes a modular collaboration strategy where autonomous agents specialize in perception (seed generation), reasoning (vulnerability analysis), and decision-making (robustness assessment). Crucially, we transcend standard RAG by constructing an Industrial Security Knowledge Graph (ISKG). This structured semantic base, combined with QLoRA fine-tuning, enables the agents not just to retrieve information but to reason over protocol states and dynamically evolve their testing strategy through a closed-loop learning mechanism. This design ensures that the agents remain lightweight (efficient) while grounding their generation in verifiable facts (trustworthy). The primary contributions of this paper are as follows:

- We propose MALF, a multi-agent cognitive architecture that resolves key IIoT fuzzing challenges by decomposing the complex task into collaborative processes. This specialization prevents context dilution and significantly enhances the effectiveness of vulnerability discovery.
- We introduce a Graph-Enhanced Retrieval mechanism powered by an ISKG and protocol-aware QLoRA tuning. This synergy ensures trustworthiness by mitigating LLM hallucinations through structured constraints and achieves efficiency by aligning the model with hexadecimal feature spaces for low-latency generation. Furthermore, a self-evolving knowledge loop refines mutation strategies based on real-time feedback.
- We establish a rigorous benchmark in a heterogeneous industrial attack-defense range. MALF outperforms state-of-the-art baselines (including RL-based and domain-specific methods) in terms of coverage and efficiency. A comprehensive ablation study across five degraded variants—including a critical text-based RAG baseline—isolates the individual contributions of CoT reasoning, QLoRA tuning, Knowledge Evolution, and multi-agent collaboration, providing controlled-testbed evidence for the value of structured graph knowledge over unstructured retrieval. Notably, the framework identified 14 previously unknown vulnerability candidates, including four CNVD-confirmed zero-day vulnerabilities (CNVD-2024-16009, CNVD-2025-22875, CNVD-2025-29811, CNVD-2026-06041) and 10 additional candidates under vendor review, validating its practical efficacy in safeguarding critical IIoT infrastructure.

## 2. Related Works

**Figure 1:**
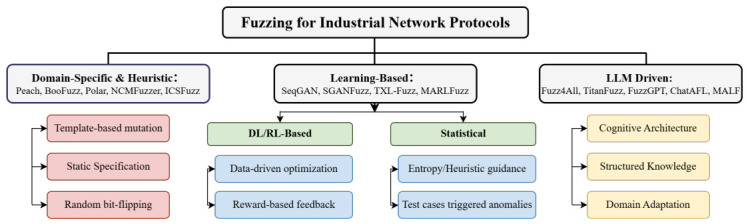
The evolutionary taxonomy of ICPs fuzzing techniques.

The landscape of vulnerability discovery for the IIoT has evolved significantly from stateless, random mutation to semantic-aware, data-driven intelligence. As illustrated in Figure 1, we categorize the evolutionary trajectory of ICP fuzzing into three distinct stages based on their level of agency and cognitive capabilities: (1) rule-based relying on static heuristics, (2) learning-based driven by Deep Learning and Reinforcement Learning, and (3) the emerging LLM-Driven Agent. This section critically reviews these paradigms to identify the specific gaps in effectiveness, efficiency, and trustworthiness that necessitate the development of our proposed framework.

### 2.1. Domain-Specific and Heuristic Fuzzing in IIoT

Traditional fuzzing in the IIoT domain has evolved from stateless random mutation to state-aware strategies coupled with protocol specifications. Early frameworks like Peach [7] and BooFuzz [12] utilized static templates to model protocol structures. To reduce manual modeling efforts, recent works such as IPSpex [13] and Polar [14] leverage traffic analysis to extract semantic fields, improving effectiveness compared to black-box methods. A notable advancement is NCMFuzzer [15], which employs information entropy to identify high-variance fields. Similarly, ICSFuzz [16] and FieldFuzz [17] utilize static analysis and reverse engineering to mine status codes from proprietary runtimes. However, these methods act as rule-based agents with limited adaptability. While entropy metrics can identify where to mutate, they lack the cognitive reasoning to determine how to mutate semantically. Consequently, they struggle to navigate the complex state machines of modern IoT devices, leading to shallow coverage and limited capability to expose deep-state vulnerabilities.

### 2.2. Deep Learning and RL-Based Fuzzing

To overcome the rigidity of heuristics, researchers have developed data-driven learning agents. Generative Adversarial Networks (GANs), such as SeqGAN [18] and SGANFuzz [19], utilize adversarial training to learn protocol distributions, while TXL-Fuzz [20] improves long-sequence modeling via Transformers. In the domain of Reinforcement Learning (RL), MARLFuzz [21] pioneered a multi-agent framework where agents cooperate to fuzz protocols like Modbus. However, RL-based methods inherently suffer from the “sparse reward” problem—agents must blindly explore vast action spaces before receiving feedback—and optimize for numerical rewards rather than semantic validity, lacking the interpretability required for trustworthy security auditing [22].

### 2.3. LLM-Driven Fuzzing Techniques

The advent of LLMs has introduced cognitive agents capable of generative reasoning. Tools like Fuzz4All [23], TitanFuzz [24], and FuzzCode [25] leverage LLMs to generate inputs via auto-prompting, while ChatAFL [26] uses LLMs to guide protocol state transitions in settings where message grammars and response feedback can be represented as text. However, deploying off-the-shelf LLM agents in IIoT presents challenges. As noted in [27] and [28], generic LLMs suffer from severe “hallucinations” when generating binary data. Unlike natural language, ICPs tolerate zero ambiguity, and existing approaches lack structured domain knowledge bases, resulting in a Knowledge Gap that wastes computational resources on invalid test cases.

## 3. Methodology

This section details MALF’s architecture, beginning with the threat model and multi-agent paradigm, followed by the ISKG construction and QLoRA-based cognitive alignment.

### 3.1. Preliminaries and Threat Model

Industrial IoT (IIoT) networks are characterized by strict real-time constraints, heterogeneous device interoperability, and resource-constrained edge environments. In this study, we elevate the conventional black-box assumptions by formalizing an adversary A operating under a grey-box (partial knowledge) paradigm, which accurately reflects the capabilities of modern Advanced Persistent Threats (APTs) targeting Cyber–Physical Systems (CPS).

We assume A possesses network access to the target IIoT environment and exhibits the following partial knowledge constraints:

Information Asymmetry (What A Lacks): A does not possess “White-Box” privileges. They lack access to PLC firmware binaries, source code, internal memory maps, or fully documented specifications of proprietary vendor-specific protocol extensions (e.g., undocumented Function Codes).

Reconnaissance Capabilities (What A Knows): A has access to publicly available vendor manuals, standard protocol specifications (e.g., standard CIP or Modbus routing headers), and the ability to passively capture legitimate baseline traffic (PCAPs) between engineering workstations and edge controllers.

Under this grey-box model, A’s objective is to synthesize fragmented, partial knowledge to bypass surface-level syntax filters and exploit deep-state protocol logic. The specific capabilities include:

- Stateful Command Injection: Correlating captured traffic and manual specifications to inject malformed, out-of-sequence, or semantic-violating packets that disrupt internal state logic without being immediately dropped by the network interface.
- Denial of Service (DoS): Exhausting PLC resources (e.g., connection pools, CPU cycles) through sequence-dependent resource hanging or high-frequency requests.
- Replay Attacks: Capturing and retransmitting valid traffic with targeted payload mutations to trigger unintended physical actions.

Information Boundary for ISKG Construction. To formalize the grey-box adversary, we partition the information available to the ISKG construction pipeline into three disjoint categories. (i) Public static specifications: vendor manuals, standard protocol documentation, and public advisories. (ii) Passive baseline traffic: PCAP sessions captured from legitimate engineering-workstation/PLC communications before the fuzzing campaign begins, without privileged access to firmware binaries, internal memory maps, or device-debugging interfaces. (iii) Evaluation outcomes: crash labels, anomaly scores, and vulnerability annotations generated during the fuzzing campaign. The ISKG ingests only categories (i) and (ii); category (iii) is consumed exclusively by the FAA’s self-evolving knowledge loop and never re-injected into the QLoRA training corpus. The ISKG therefore encodes observable communication behavior, not hidden implementation details. Because the passive baseline traffic in (ii) was collected from devices belonging to the same laboratory deployment as the evaluation targets, the resulting setup is properly characterized as a testbed-informed grey-box scenario rather than a fully external adversary; an external attacker would need equivalent passive access to reproduce the ISKG construction step. We restate this caveat in the Limitations paragraph of Section 5.

Unlike conventional IT protocols, Industrial Control Protocols (ICPs, e.g., Modbus/TCP, S7Comm) often lack native robust authentication or encryption. Therefore, our methodology specifically focuses on identifying deep-state implementation flaws in packet parsing, state management, and resource allocation that allow a grey-box adversary to achieve these exploitations.

### 3.2. Framework Overview

**Figure 2:**
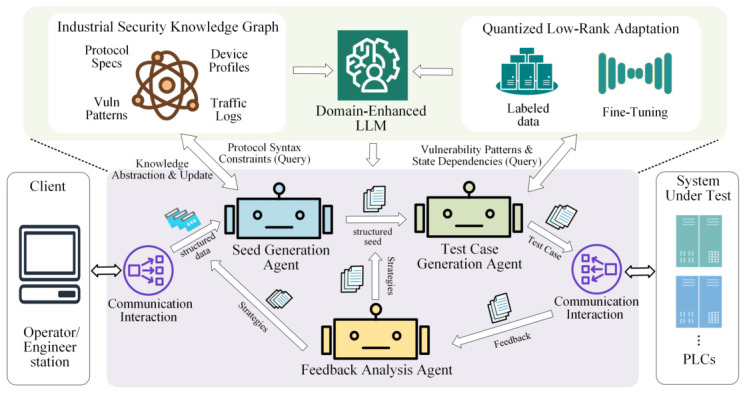
The framework architecture of MALF.

To automate vulnerability discovery within this threat landscape, we propose MALF (Multi-Agent LLM Fuzzing Framework). As illustrated in Figure 2, MALF departs from monolithic fuzzing architectures by employing a knowledge-driven multi-agent system. The framework is centered around a shared, dynamic ISKG, which serves as the semantic substrate for three specialized cognitive agents and an asynchronous coordination layer:

- Seed Generation Agent (SGA): Responsible for reconstructing high-quality, protocol-compliant initial seeds. It queries the ISKG to map raw traffic bytes to semantic protocol states, ensuring syntactic validity and reducing the search space for subsequent mutations.
- Test Case Generation Agent (TGA): Tasked with the exploitation phase. It functions as a conditional generator, leveraging vulnerability patterns retrieved from the ISKG to execute Intent-Driven Mutations (e.g., specific state logic violations or boundary value attacks) rather than random bit-flipping.
- Feedback Analysis Agent (FAA): Acts as the “Critic” and “Learner.” It evaluates SUT responses to optimize mutation strategies in real-time. Crucially, it abstracts novel anomaly patterns from execution logs and injects them back into the ISKG, realizing a self-evolving knowledge loop that enhances the trustworthiness of future generations.
- Communication Interaction Module: A synchronization layer based on the ZeroMQ protocol that orchestrates the asynchronous data flow between agents and the SUT, ensuring high-throughput parallel execution.

By grounding agent cognition in the ISKG and refining it via QLoRA, MALF combines graph-based structured reasoning with neural generative capabilities.

### 3.3. Technical Foundations

**Figure 3:**
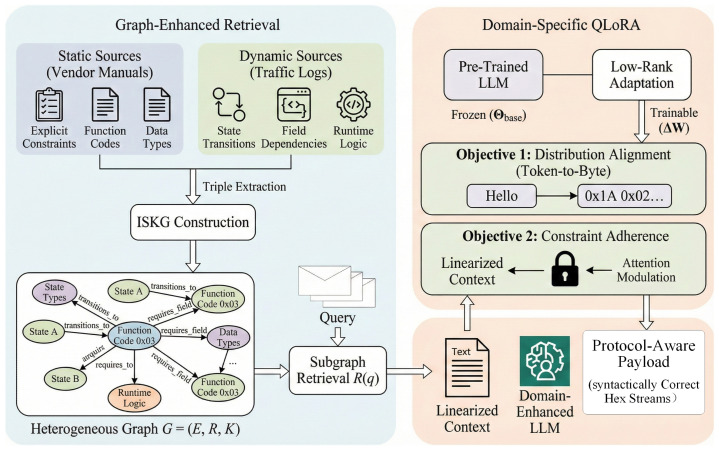
Technical architecture of the multi-agent LLM: ISKG and QLoRA pipelines.

The efficacy of intelligent fuzzing hinges on the model’s ability to bridge the gap between high-level protocol logic and low-level byte generation. MALF addresses this through a dual-pillar architecture: a knowledge graph for structured knowledge injection and domain-specific instruction tuning via QLoRA for cognitive alignment. The technical architecture is shown in Figure 3.

#### 3.3.1. Graph-Enhanced Retrieval

While RAG relies on heuristic chunking of unstructured text, ICPs demand strict structural adherence. We propose constructing an ISKG to serve as the structured knowledge base for our retrieval mechanism. This approach upgrades the retrieval target from disjointed text segments to interconnected semantic triples, enabling the agents to reason over complex protocol dependencies [29]. We define ISKG as a heterogeneous graph G=(E,R,K), fusing knowledge from two complementary dimensions:

- Static Specification Modeling (Specification-Driven): Deriving from MALF’s document parsing pipeline, we extract explicit protocol constraints directly from vendor manuals. These rigid rules form the skeletal structure of the graph, ensuring the generated seeds strictly adhere to the standard protocol definitions.
- Dynamic Behavior Integration (Data-Driven): Recognizing that real-world implementations often diverge from static documentation, we enrich the ISKG with behavioral insights derived from actual traffic logs. Rather than relying solely on manuals, we incorporate observed state transitions and field dependencies from historical communication traces. These behavioral patterns are mapped to dynamic triples ($\langle State_{A},\mathrm{transitionsTo},State_{B}\rangle$) within the graph. This integration ensures the agents operate on a “living” knowledge base that reflects the protocol’s practical runtime logic, without requiring the framework to innovate on protocol reverse engineering algorithms itself.

For Static Specifications, an LLM-assisted NLP pipeline parses vendor manuals and extracts semantic entities into (Head,Relation,Tail) triples, audited by an ICS security expert (one-time setup, 24–48 h per protocol). For Dynamic Behaviors, a Scapy-based script ingests PCAP traffic logs, performs session tracking, and infers state-machine transitions. For instance, if Forward_Open consistently precedes Write_Data, the script generates the temporal triple 〈State_Forward_Open, MUST_PRECEDE, State_Write〉. The resulting ISKG is persistently stored in a Neo4j graph database, while an in-memory NetworkX projection is utilized during the fuzzing phase to ensure sub-millisecond retrieval latency. The scale of the ISKG varies with protocol complexity: the Modbus/TCP subgraph comprises approximately 280 triples, whereas the S7Comm and CIP subgraphs scale to over 900 triples each.

By synthesizing static specifications with dynamic traffic insights, the ISKG provides a comprehensive semantic substrate. During fuzzing, the retrieval module R(q) queries this graph to extract relevant subgraphs, which are then linearized into structured context for the LLM.

ISKG Construction Reproducibility. For full reproducibility, we summarize the construction workflow used in the present study. (1) Source documents: Per protocol, we collected vendor-published manuals (Siemens SIMATIC S7-300/1200 manuals, Rockwell Logix Communication and CIP Network Library, Schneider Modicon programming guides, Mitsubishi MELSEC iQ-F user manuals, Omron CX-Programmer manuals, and the official Modbus, EtherNet/IP-CIP, OPC UA, and FINS specifications) together with relevant public CVE/CNVD advisories. (2) LLM-assisted parser: The documents were segmented section-by-section and processed by a Qwen-3-8b-based extractor with a fixed prompt template that emits triples in the form (Head,Relation,Tail), restricted to a closed vocabulary of nine relation types (has_field, has_constraint, precedes, follows, requires_state, maps_to_response, has_opcode, is_subtype_of, and vul_to). (3) Expert validation workflow: Every extracted triple was manually reviewed by two ICS security experts, who independently labeled each triple as accept, reject, or revise; disagreements were resolved by consulting the source manual and, when needed, replaying the corresponding PCAP trace. The pre-arbitration percent agreement between the two experts was 92.4% across the five evaluated protocols, and only triples accepted by both experts after arbitration were retained. (4) PCAP-derived dynamic transitions: A Scapy-based session tracker ingested the passive baseline PCAPs (Section 3.3.2), grouped packets into protocol sessions by five-tuple and protocol-specific session identifiers, and emitted temporal triples $\langle State_{A},\mathrm{precedes},State_{B}\rangle$ whenever a transition was observed at least three times across distinct sessions, in order to suppress one-off anomalies. (5) Neo4j ingestion with consistency checks: Validated triples were imported into Neo4j 5.x with uniqueness constraints on (Head,Relation,Tail), and a Cypher-based linter rejected duplicate triples, dangling references, and relations that violated the typed schema. End-to-end, the one-time setup required approximately 24–48 h of expert effort per protocol (10–14 h for document parsing and triple extraction, 10–20 h for expert validation, and 4–8 h for PCAP-based dynamic-transition verification).

#### 3.3.2. Domain-Specific Instruction Tuning via QLoRA

Qwen-3-8b provides strong general reasoning, but its probability distribution is aligned with natural language rather than hexadecimal byte streams. We employ QLoRA [30] to bridge this modal gap, treating fuzzing as a conditional generation problem P(Payload|Context,Intent). QLoRA freezes the pre-trained weights $\Theta_{base}$ and injects trainable low-rank adapter matrices ΔW:

(1) $$h=W_{base}x+\lambda W_{A}W_{B}x$$

(2) $$L=-\sum_{i}\log P\left(y_{i}|x_{i};\Theta_{\mathrm{base}},W_{A}W_{B}\right),$$

where $W_{A}\in R^{d\times r}$ and $W_{B}\in R^{r\times d}$ encode the task-specific features with rank r≪d, $\Theta_{\mathrm{base}}$ represents the frozen parameters of the pre-trained model, $x_{i}$ represents the input, and $y_{i}$ represents the target output. By optimizing the adapter weights ΔW on this structured dataset, we achieve three critical objectives:

- Distribution Alignment (Token-to-Byte Shift): The pre-trained model inherently favors natural language fluency. Through instruction tuning on a corpus of valid protocol traces, we align the model’s output distribution with the hexadecimal feature space of industrial protocols. The optimization minimizes the divergence between the model’s predicted token probabilities and actual valid protocol bytes. This ensures that the model learns to prioritize valid control characters and delimiters over linguistically plausible but protocol-invalid tokens.
- Constraint Adherence (Attention Modulation): A core challenge in LLM-driven fuzzing is “hallucination,” where the model ignores provided constraints. Our QLoRA tuning specifically reinforces the model’s ability to attend to the RAG-retrieved Context. By training on pairs of (Structured_Constraints, Valid_Payload), the adapter weights ΔW learn to modulate the self-attention mechanism, effectively treating the retrieved knowledge (e.g., “Length must be 2 bytes”) as hard constraints rather than optional context. This significantly reduces the generation of syntactically correct but semantically rejected packets.
- Token-to-Byte Alignment: A critical challenge in deploying generic LLMs for binary protocol fuzzing is the tokenization clash. Standard BPE tokenizers are optimized for natural language and handle continuous hexadecimal streams unpredictably, causing severe token fragmentation that destroys spatial alignment. To resolve this, we implement a strict 1-to-1 byte-to-token mapping. First, we expand the Qwen tokenizer’s vocabulary by adding 256 dedicated special tokens (<0x00> through <0xFF>) and resize the embedding matrix accordingly. Second, all raw traffic is pre-processed into space-delimited hex pairs (e.g., 0A 1B 2C) to govern the tokenizer’s parsing boundaries. The QLoRA fine-tuning was conducted on a proprietary dataset comprising 85,000 instruction–response pairs, automatically synthesized from 120 h of PCAP traffic captured within our testbed. The dataset spans diverse protocols (30% Modbus/TCP, 25% S7Comm, 25% EtherNet/IP, 10% SRTP, and 10% HTTP). Because typical IIoT payloads range from 15 to 150 bytes, this 1-to-1 mapping keeps the average sequence length efficient (≈450–600 tokens per prompt, including CoT context), enabling the model to learn precise byte-level probability distributions.

This tuning paradigm enables MALF to acquire domain-specific generation patterns while retaining the generalization capabilities of a large language model.

Training and Evaluation Separation. To reduce device-level data leakage, QLoRA fine-tuning and evaluation were separated at the PLC-model level. PCAP sessions used to synthesize the 85,000 instruction–response pairs were collected exclusively from a designated training subset of devices (Siemens S7-1200, Munich, Germany; Rockwell 1766-L32BWA, Milwaukee, WI, USA; Mitsubishi FX5U-32MR/ES, Tokyo, Japan; Schneider TM221CE16T, Rueil-Malmaison, France; and Omron CP2E-N14RDR-A, Kyoto, Japan). Evaluation was conducted on a strictly disjoint held-out subset that includes Siemens S7-300, Munich, Germany; Rockwell 1769-L30ER and 1789-L60 SoftLogix, Milwaukee, WI, USA; Emerson VersaMax IC200, St. Louis, MO, USA; Inovance EZ320-0808TP, Shenzhen, China; Panasonic SFDOC32ET, Tokyo, Japan; Phoenix MAX100-24T, Blomberg, Germany; and Delta AS228T-A, Taipei, China. No PCAP session captured from a held-out device entered the QLoRA training corpus, and the split was fixed before any fuzzing experiment was launched. Because some passive baseline traffic and the held-out devices originate from the same laboratory testbed, we interpret the resulting setting as a testbed-informed grey-box scenario rather than as a fully device-independent generalization guarantee; we discuss this scope in Section 5.

### 3.4. Multi-Agent Fuzzing Workflow

The core innovation of MALF lies in decomposing the monolithic fuzzing process into a collaborative workflow driven by specialized agents. This section formalizes the cognitive processes of the Seed Generation and Test Case Generation Agents, detailing how they leverage the ISKG and QLoRA-tuned LLM to achieve protocol-aware fuzzing.

#### 3.4.1. Knowledge-Guided Reconstruction

The SGA functions as a semantic reconstructor, tasked with transforming unstructured network traffic into high-quality, protocol-compliant seed corpora. Unlike traditional fuzzers that rely on bit-level recording, the SGA utilizes Chain-of-Thought (CoT) [31] reasoning to reconstruct seeds that satisfy both syntactic and semantic constraints retrieved from the ISKG.

**Figure 4:**
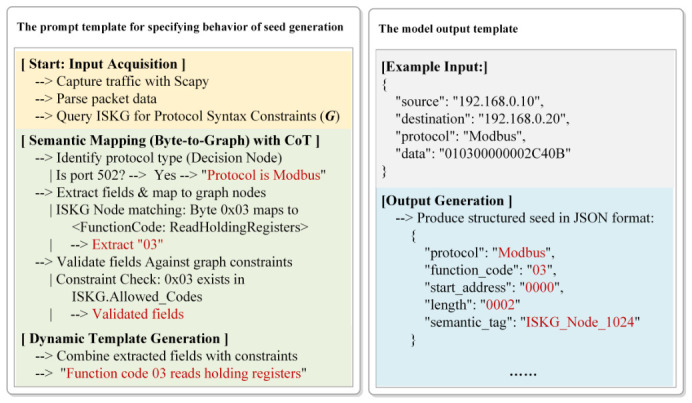
Prompt template for specifying behavior of seed generation.

The reconstruction process is modeled as a mapping function $F:T_{raw}\times G\to S_{struct}$, where $T_{raw}$ is the captured traffic stream and *G* is the ISKG. The SGA executes this in three phases:

1. State-Message Mapping: Leveraging the dynamic behavioral insights integrated into the ISKG, the agent first maps a captured payload $p_{i}$ to a specific protocol state $\tau_{j}$ (e.g., Modbus_Write_Multiple_Registers).
2. Constraint Retrieval: The agent queries the ISKG to retrieve the semantic ontology associated with $\tau_{j}$. For instance, extracting valid ranges and dependencies:
  (3) $$C_{\tau }=\left\{\left(e,r,v\right)\in K|e=\tau_{j},r\in \left\{\mathrm{hasField},\mathrm{hasValidRange}\right\}\right\},$$
3. CoT-Driven Instantiation: As illustrated in Figure 4, the agent employs a CoT prompt strategy to progressively validate fields. The QLoRA-tuned model generates the structured seed $s_{j}$ by solving
  (4) $$s_{j}=\arg\max_{s}P\left(s|p_{i},C_{\tau };\Theta_{tuned}\right),$$

This formulation ensures that the generated seed $s_{j}=\langle \mathrm{FC}=0x10,\mathrm{Addr}∼U\left[0,100\right],\ldots \rangle$ strictly adheres to the vendor-specific constraints defined in $C_{\tau }$, significantly reducing the rejection rate during the initial handshake. The quantitative impact of CoT reasoning is isolated in the ablation study (Section 4.4, Direct-Gen variant), where removing CoT reduces TCAR by 12.0 percentage points and the total vulnerability count by 55.2% (from 29 to 13 unique findings on the ablation subset).

#### 3.4.2. Vulnerability-Pattern Driven Mutation

The TGA operates as the exploitation engine. Unlike random mutation strategies, the TGA performs Intent-Driven Mutation. It treats fuzzing as a conditional generation task, where the generation of a test case *t* is conditioned on the seed *s* and specific vulnerability patterns κ retrieved from the ISKG. We define the mutation probability distribution as

(5) $$t∼P_{\phi }\left(t|s,\kappa \right),\;\mathrm{where}\;\kappa =R_{ISKG}\left(\mathrm{Vul\_Patterns},s\right),$$

Here, κ represents abstract attack patterns (e.g., Connection_Flooding or Length_Overflow) linked to the target protocol in the ISKG. The TGA utilizes the QLoRA-tuned LLM to interpret these high-level patterns and apply targeted mutation operators.

**Figure 5:**
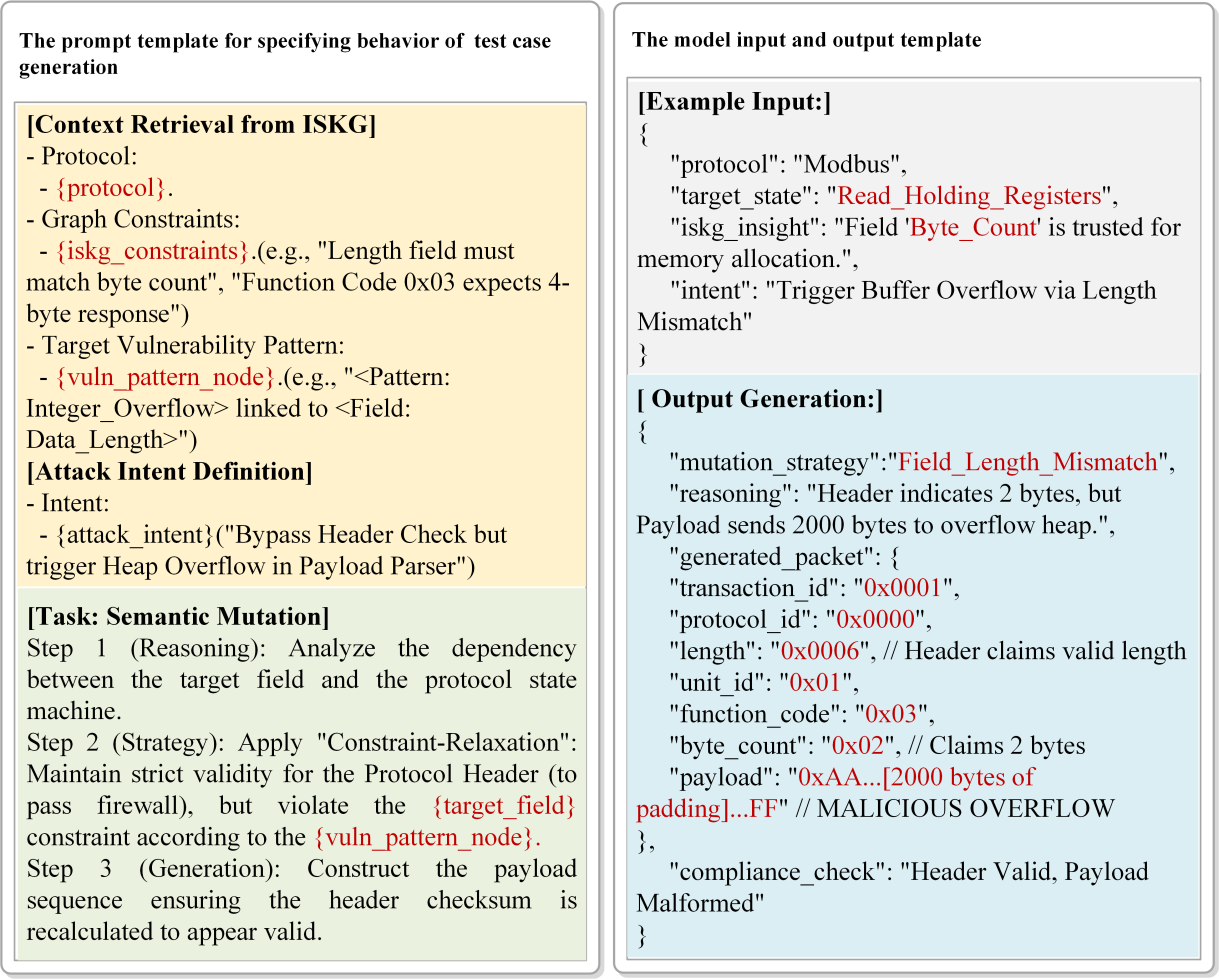
Structured prompt template for the Test Case Generation Agent (TGA), showing the agent role, required inputs, reasoning steps, and expected output format.

Guided by the dynamic prompt template shown in Figure 5, the TGA decomposes the mutation task into structured CoT steps. This corresponds to the execution logic described in Algorithm 1. The agent utilizes retrieved constraints C and specific intents I to select among three distinct classes of operators.


**Algorithm 1** Intent-Driven Mutation via ISKG and QLoRA
**Require:** Seed *s*, ISKG *G*, QLoRA-tuned Model $M_{\theta }$**Ensure:** Mutated Test Case *t*  1:
**Initialization:**
  2:

p←s.protocol

  3:

t← *⌀*
  4:{**Phase 1: Knowledge Retrieval**}  5:*κ* ← GetPatterns(G,p) {Retrieve Vuln. Patterns}  6:C← GetConstraints(G,p) {Retrieve Dependencies}  7:{**Phase 2: Intent Formulation**}  8:$I\leftarrow M_{\theta }.$ Reason(s,κ) {CoT-based intent analysis}  9:{**Phase 3: Dynamic Prompt Construction**}10:

P←{Role:Expert,Context:C,Seed:s,Intent:I}

11:
**Switch**  I  **do**
12:**if** I  targets *Boundary Values* **then**13:   $P.\mathrm{addTask}(“\mathrm{Field-Level}\;\mathrm{using}\;B_{ISKG}”)$14:**else if**  I targets *Parser Logic* **then**15:   P.addTask(“Structural(Insert/Delete)”)16:
**else**
17:   P.addTask(“Semantic(ViolateConstraints)”)18:
**end if**
19:{**Phase 4: Conditional Generation**}20:

$t\leftarrow M_{\theta }.$ Generate (P)

21:
*// Output: $v^{\prime }=v+\delta$ or $P^{\prime }=P\cup F_{ins}$ or $S_{sem}^{\prime }$*
22:**return** *t*



The three mutation operator classes are formalized as follows:

Field-Level Mutation (Boundary Probing): Targeting specific fields identified by constraint relations in the ISKG. For a field value *v*, the mutation is defined as

(6) $$v^{\prime }=v+\delta ,\;\mathrm{where}\;\delta \in B_{ISKG}\cup N\left(0,\sigma \right),$$

Here, $B_{ISKG}$ denotes the set of critical boundary values extracted from the graph, and N represents stochastic noise.

Structural Mutation (Parser Stressing): Designed to test parser robustness by altering the Protocol Data Unit (PDU) topology. The structural transformation is formalized as

(7) $$P^{\prime }=\left(P\cup F_{\mathrm{insert}}\right)\setminus F_{\mathrm{delete}},$$

The choice of $F_{\mathrm{insert}}$ (illegal fields) and $F_{\mathrm{delete}}$ (mandatory fields) is non-random; it is guided by ISKG knowledge regarding “Required Fields” for specific Function Codes, prioritizing the removal of fields essential for state validation.

Semantic Mutation (Logic Violation): This operator introduces logical inconsistencies to violate protocol state integrity. Defined as a transformation function M:

(8) $$S_{semantic}^{\prime }=M\left(s\right)|\exists \left(f_{i},f_{j}\right)\in s,\mathrm{Constraint}\left(f_{i},f_{j}\right)\;\mathrm{is}\;\mathrm{False},$$

For example, if the ISKG asserts a dependency Length = sizeof(Payload), the TGA intentionally constructs a packet where Length≠sizeof(Payload) to trigger buffer read anomalies.

 
**Remark 1.** 
*Equations  (6)–(8) define the discrete action space of the TGA, not optimization objectives. The QLoRA-tuned LLM acts as a semantic optimizer, selecting operators via intent analysis (I) and ISKG constraints (C), thereby transforming static heuristics into intent-driven exploitation*.

#### 3.4.3. Adaptive Strategy and Knowledge Evolution

The FAA serves a dual purpose: acting as a Real-Time Critic that modulates immediate testing parameters, and a Knowledge Curator that drives the long-term evolution of the ISKG. This agent closes the Testing–Discovery–Learning loop, ensuring that the system does not merely detect vulnerabilities but learns from them.

**Figure 6:**
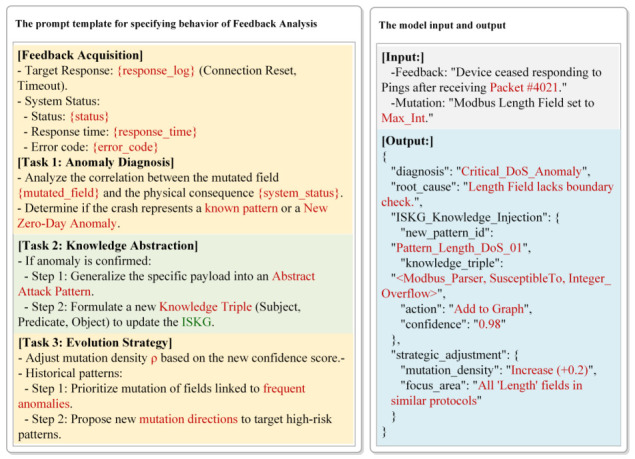
Structured prompt template for the Feedback Analysis Agent (FAA), showing response interpretation, anomaly scoring, replay verification, and feedback-generation logic.

Metric Quantification: To standardize the diverse responses from the system under test (SUT), the FAA employs a weighted scoring mechanism. As depicted in Figure 6, the agent processes the raw response vector to compute a scalar anomaly score *S*:

(9) $$S=w_{1}\cdot E_{type}+w_{2}\cdot T_{latency}+w_{3}\cdot R_{resource},$$

where $E_{type}$ quantifies the error category and $w_{i}$ are configurable weights based on the testing objective.

Dynamic Strategy Modulation: Based on *S*, the agent acts as a controller in a feedback control loop, dynamically adjusting the mutation density ρ for the TGA. The update rule is governed by

(10) $$\rho_{t+1}=\rho_{0}\cdot \left(1+\beta \cdot \frac{S}{S_{\max}}\right),$$

where β is a scaling factor. This ensures a transition from broad exploration to focused exploitation when high-risk anomalies are detected.

Knowledge Abstraction and Graph Evolution: Crucially, beyond parameter tuning, the FAA implements a Knowledge Abstraction mechanism. When a critical anomaly ($S>\tau_{critical}$) is detected, the agent triggers an abstraction function A to distill the effective test case *t* and its consequence $r_{exception}$ into a structured semantic triple, we denote the newly discovered vulnerability pattern as $\kappa_{new}$:

(11) $$\Delta K=A\left(t,r_{exception}\right)\to \langle \mathrm{TargetDevice},\mathrm{vul\_To},\kappa_{new}\rangle ,$$

For instance, if a specific sequence *t* causes an “I/O Module Stoppage” (as observed in our S7-300 experiments), the agent abstracts this into a formal vulnerability pattern. This new knowledge is injected into the graph, updating the system’s ontology:

(12) $$G_{k+1}=G_{k}\cup \Delta K,$$

This update process enables the TGA to immediately utilize $\kappa_{new}$ for targeted cross-protocol testing, realizing a self-evolving fuzzing capability.

Deterministic Crash Verification: In physical ICS environments, anomalies such as connection resets or timeouts may result from transient network congestion rather than exploitable flaws. To mitigate false positives, the FAA implements a two-stage verification protocol. First, when a critical anomaly ($S>\tau_{critical}$) is detected, the triggering test case is replayed three consecutive times against a freshly reset PLC state; the anomaly is retained only if it reproduces deterministically on all three replays. Second, the FAA performs an out-of-band heartbeat check via ICMP ping and a safe diagnostic read: if the PLC drops the malformed packet but responds to the valid probe, it is classified as robust error handling rather than a crash. This mechanism filtered 58 initial anomaly flags to 29 confirmed unique vulnerabilities (14 zero-day candidates, 15 N-Day), achieving a post-verification False Positive Rate of 25.4% across the full evaluation campaign.

FAA Parameter Configuration. For full reproducibility, Table 1 reports the values of the FAA scoring weights, the critical anomaly threshold, the normalization constant, and the mutation-density scaling factor used in all reported experiments. The same parameter set was applied uniformly across the five evaluated protocols and was fixed prior to the comparative evaluation; no protocol-specific tuning was performed after observing test outcomes.

#### 3.4.4. Communication Interaction Module

To support the high-throughput demands of industrial fuzzing, the Communication Interaction Module implements a decoupled, event-driven architecture. We address the challenges of parallelization and fault tolerance through a ZeroMQ-based Publish/Subscribe (PUB/SUB) topology [32], as detailed in Algorithm 2.

The coordination mechanism transforms the linear fuzzing process into asynchronous data streams:

- Seed Stream ($Q_{seed}$): The SGA operates as a Publisher, broadcasting structured seeds via the $Q_{seed}$ channel. The TGA subscribes to this stream, allowing seed generation to proceed independently of mutation latency.
- Execution Stream ($Q_{exec}$): The TGA publishes mutated payloads (test cases *t*) to $Q_{exec}$, which are consumed by the parallel execution workers connected to the physical SUT.
- Feedback Stream ($Q_{fb}$): Responses *r* are published to $Q_{fb}$, enabling the FAA to process results asynchronously without blocking the generation engine.

This architecture inherently supports horizontal scalability. As shown in the Daemon process of Algorithm 2, the module actively monitors agent health. We denote the physical timestamp as τ. The status of an agent $a_{i}$ is defined as

(13) $$\mathrm{Status}\left(a_{i}\right)=\left\{\begin{array}{cc} \mathrm{Active}, & \mathrm{if}\;\tau_{now}-\tau_{last}\left(a_{i}\right)<\delta_{timeout},\\ \mathrm{Failed}, & \mathrm{otherwise}. \end{array}\right.$$

In the event of an agent failure, the system automatically redistributes the workload by re-routing the ZeroMQ topics to healthy instances. This fault-tolerant design ensures continuous operation during long-duration (24 h+) fuzzing campaigns. We note that ZeroMQ channels operate within an isolated laboratory network; in production deployments, CurveZMQ encryption would be applied to mitigate additional attack surface concerns.


**Algorithm 2** Asynchronous Coordination via ZeroMQ
**Require:** ZeroMQ Context Z, Agents Set A**Ensure:** Continuous Fuzzing Data Flow  1:
**Pipeline Initialization:**
  2:$Q_{seed}\leftarrow Z.$Socket(PUB) {Stream 1: SGA → TGA}  3:$Q_{exec}\leftarrow Z.$Socket(PUB) {Stream 2: TGA → Workers}  4:$Q_{fb}\leftarrow Z.$Socket(PUB) {Stream 3: SUT → FAA}  5:
**Process 1: Seed Generation Agent (SGA)**
  6:    **while** True **do**  7:    s←CaptureTraffic()  8:    $Q_{seed}.$Publish(“topic_seed”,s)  9:    SendHeartbeat$\left(ID_{SGA}\right)$10:
**Process 2: Test Case Generation Agent (TGA)**
11:   $Q_{seed}.$Subscribe(“topic_seed”)12:   **while** True **do**13:     $s\leftarrow Q_{seed}.$Receive()14:     t← Alg1_Generate(s)15:     $Q_{exec}.$Publish(“topic_exec”,t)16:
**Process 3: Execution Worker (SUT Interface)**
17:   $Q_{exec}.$Subscribe(“topic_exec”)18:   **while** True **do**19:     $t\leftarrow Q_{exec}.$Receive()20:     r← ExecuteOnSUT(t)21:     $Q_{fb}.$Publish(“topic_fb”,r)22:
**Process 4: Feedback Analysis Agent (FAA)**
23:   $Q_{fb}.$Subscribe(“topic_fb”)24:   **while** True **do**25:     $r\leftarrow Q_{fb}.$Receive()26:    Update ISKG(r) {See Equation (12)}27:
**Daemon: Health Monitor**
28:   **for each** $a_{i}\in A$ **do**29:     $\Delta \tau \leftarrow \tau_{now}-\tau_{last}\left(a_{i}\right)$ {Time check}30:     **if** $\Delta \tau >\delta_{timeout}$ **then**31:       RedistributeTasks$\left(a_{i}\right)$32:       RestartContainer$\left(a_{i}\right)$33:     **end if**34:   **end for**



### 3.5. Deployment Model

A key distinction exists between MALF’s offline training and online inference phases. The QLoRA fine-tuning is conducted on a high-performance cluster (8 × H100 GPUs) as a one-time offline process. However, the 8B-parameter model, when loaded at 4-bit precision via QLoRA, requires only 6–8 GB VRAM for inference. Consequently, the online fuzzing phase operates on consumer-grade hardware (a single RTX 3060 or NVIDIA Jetson AGX Orin suffices per agent instance). In our evaluation, a workstation with dual RTX 4090 GPUs ran four parallel TGA workers to maximize throughput, not because a single model demands such resources. This separation ensures that MALF’s online inference is deployable at the industrial edge while the heavy training cost is amortized offline.

## 4. Evaluation

To rigorously validate the proposed framework, we answer the following Research Questions (RQs):

- RQ1: Does MALF demonstrate statistically significant superiority in vulnerability discovery efficiency and probability compared to state-of-the-art (SOTA) methods?
- RQ2: Does the self-evolving knowledge loop actively improve mutation strategies over time, evidencing a “learning” capability?
- RQ3: Can MALF generalize across heterogeneous vendors to identify zero-day vulnerabilities in diverse real-world IIoT environments?

### 4.1. Experimental Setup

#### 4.1.1. Computational Environment

The offline QLoRA fine-tuning was performed on a cluster equipped with eight NVIDIA H100 GPUs and Intel Xeon Gold 4310 CPUs (12C/24T). The online fuzzing deployment was hosted on a workstation with two NVIDIA RTX 4090 GPUs (running four parallel TGA instances at 4-bit precision, ≈6–8 GB VRAM per instance) and 128 GB DDR5 memory. The software stack includes Python 3.9+, Hugging Face Transformers, and a customized Scapy layer for protocol interaction.

Training/Evaluation Device Separation. Consistent with the device-level split established in Section 3.3.2, the PLCs used for QLoRA fine-tuning are disjoint from those used in the comparative evaluation and zero-day discovery campaigns reported in Section 4.3, Section 4.4 and Section 4.5. All comparative metrics (TCAR, *H*, $C_{state}^{ISKG}$, $C_{resp}$, N-Day Discovery Yield, and TTE) and all 14 zero-day candidates were obtained on held-out devices that did not contribute traffic to the QLoRA training corpus. Reviewers can therefore interpret the reported numbers as performance on devices unseen during fine-tuning, with the residual caveat that all devices share the same laboratory testbed.

#### 4.1.2. Fuzz Testing Platform

**Figure 7:**
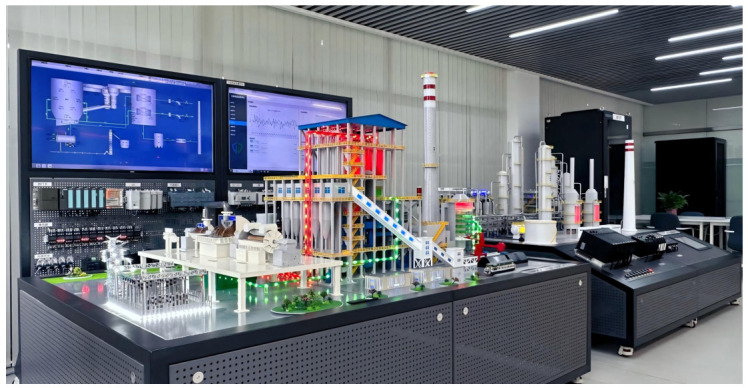
Experimental platform: the heterogeneous IIoT testbed for power plant and petrochemicals.

To evaluate the fuzzers’ generalization capabilities across diverse industrial ecosystems, we deployed the framework within a high-fidelity industrial attack-defense range as shown in Figure 7. Unlike limited simulations, this testbed integrates a heterogeneous array of PLCs, IoT sensors, and monitoring workstations, mirroring the complex interoperability of real-world CPSs.

**Figure 8:**
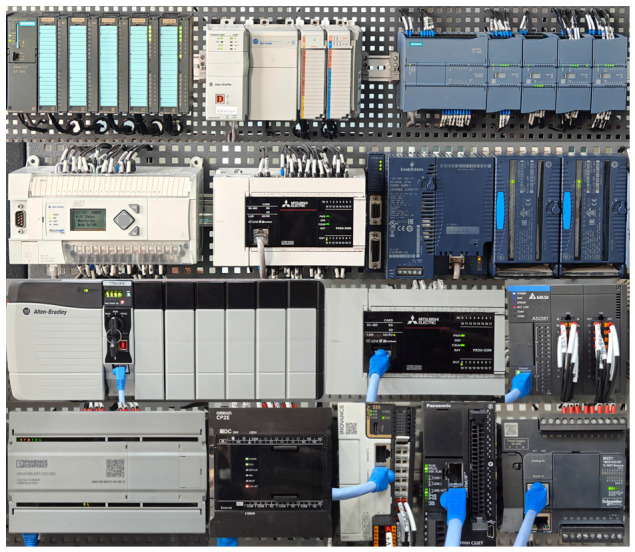
System under test equipment.

As detailed in Figure 8 and Table 2, the testing environment encompasses devices from ten major global manufacturers and supports a wide spectrum of protocols ranging from standard Modbus/TCP and EtherNet/IP to proprietary implementations like S7Comm, Melsoft, and FINS. Notably, the platform employs a hybrid architecture that combines physical hardware with industrial-grade SoftPLCs. This diverse configuration allows for a rigorous assessment of the fuzzers’ ability to navigate vendor-specific protocol dialects and identify vulnerabilities across varying hardware architectures.

#### 4.1.3. Baseline Methods

To comprehensively validate MALF’s superiority, we benchmark it against four distinct categories of state-of-the-art fuzzers, covering traditional state-aware methods, domain-specific optimizations, Reinforcement Learning approaches, and general-purpose LLM tools:

Enhanced BooFuzz [12] (Traditional State-Aware): A Python-based framework derived from Sulley, enhanced with extended process monitoring for closed-source SCADA systems. Serves as the benchmark for rule-based fuzzing without AI augmentation.

NCMFuzzer [15] (Domain-Specific SOTA): Employs Non-Critical Field Mutation combined with entropy-based field identification for specialized ICS protocol fuzzing.

MARLFuzz [21] (Multi-Agent RL): Utilizes Multi-Agent Reinforcement Learning where agents coordinate via RNNs to learn temporal and spatial message features.

Fuzz4All [23] (General LLM): A universal fuzzer utilizing auto-prompting to generate inputs for diverse systems, serving to isolate the contribution of domain-specific knowledge and multi-agent collaboration.

Note on Baseline Selection: Baselines are restricted to black-box and grey-box network protocol fuzzers consistent with our threat model. White-box source-code fuzzers (e.g., FuzzCode [25]) cannot be deployed against proprietary, closed-source IIoT devices and are thus excluded. We considered ChatAFL [26] as a close published LLM-guided grey-box protocol fuzzer to MALF. However, we did not include it as an executable baseline because its feedback loop assumes response information that can be interpreted as text-level protocol states or textual error messages. This assumption does not hold for the industrial PLC protocols in our testbed. For example, a text-oriented protocol may expose an error as an explicit string such as ERROR: ILLEGAL FUNCTION, whereas a PLC protocol often returns only compact binary status fields, such as a Modbus exception response encoded as 0x82 0x01, where 0x82 denotes the exception form of the requested Function Code and 0x01 denotes an illegal-function exception. Such byte-level replies do not provide the textual feedback expected by ChatAFL’s LLM-guided state-feedback loop. Making ChatAFL operate on these replies would require protocol-specific binary parsers, exception-code decoders, and safety wrappers for physical PLC interaction, which would constitute substantial re-engineering rather than unmodified baseline execution. We therefore exclude ChatAFL from the executable baselines and restrict empirical comparison to tools that can operate against closed-source PLCs under the same black-box or grey-box testbed constraints.

### 4.2. Evaluation Criteria

#### 4.2.1. Test Case Quality

Test Case Acceptance Rate (TCAR): Measures the semantic validity of the generated seeds.

(14) $$TCAR=\frac{N_{accepted}}{N_{generated}}\times 100\%,$$

where $N_{accepted}$ denotes test cases that pass the SUT’s packet inspection and elicit a valid application-layer response.

Shannon Entropy (*H*): Quantifies mutation diversity [33]:

(15) $$H\left(X\right)=-\sum_{i}p\left(x_{i}\right)\log_{2}p\left(x_{i}\right),$$

where $p(x_{i})$ is the probability of observing a specific byte pattern $x_{i}$ in the corpus.

ISKG-Defined State Coverage ($C_{state}^{ISKG}$): Quantifies the fraction of ISKG-encoded protocol states traversed during fuzzing:

(16) $$C_{state}^{ISKG}=\frac{|{⋃}_{i=1}^{M}\left\{S_{i,1},\ldots ,S_{i,k}\right\}|}{|S_{total}^{ISKG}|},$$

where $S_{total}^{ISKG}$ denotes the state space encoded in the ISKG. Because the denominator is graph-defined, this metric measures graph-relative protocol-state exploration rather than firmware code coverage; it does not bound exploration of states absent from the ISKG, and we therefore interpret it as evidence of ISKG-relative coverage rather than as a fully ISKG-independent guarantee. To complement this graph-relative measure, we additionally report a Response-Class Coverage metric:

(17) $$C_{resp}=\frac{|R_{obs}|}{|R_{ref}|},$$

where $R_{obs}$ is the set of distinct protocol response classes (status codes, exception codes, and observable device-response states) triggered during fuzzing, and $R_{ref}$ is a reference response set derived from passive baseline PCAPs and public protocol specifications. Because $R_{ref}$ is constructed independently of the ISKG, $C_{resp}$ provides a cross-check that is not subject to the same circularity as $C_{state}^{ISKG}$.

#### 4.2.2. Vulnerability Discovery Efficiency

To answer RQ1, we employ time-domain metrics to evaluate how efficiently MALF identifies unique vulnerabilities.

Cumulative Vulnerability Discovery (B(t)): To visualize the discovery trend, we define B(t) as the cumulative number of unique vulnerabilities detected by time *t*:

(18) $$B\left(t\right)=\sum_{i=1}^{N_{bug}}I\left(t_{i}\leq t\right),$$

where $N_{bug}$ is the total count of known vulnerabilities, and I(·) is an indicator function.

Time-to-Exposure (TTE): For each unique vulnerability $v_{i}$, we record the time elapsed before its first detection:

(19) $$TTE_{i}=t_{first\_detect}\left(v_{i}\right)-t_{start},$$

False Positive Rate (FPR): In physical ICS environments, anomalies may arise from transient network congestion rather than exploitable flaws. We define FPR as

(20) $$FPR=\frac{N_{false}}{N_{flagged}}\times 100\%,$$

where $N_{flagged}$ is the total number of unique anomalies flagged by the FAA and $N_{false}$ is the number classified as non-exploitable after deterministic replay verification (Section 3.4.3). Baseline fuzzers lack an automated crash verification mechanism; all flagged anomalies require manual triage. MALF’s autonomous false-positive filtering reduces the number of alarms requiring manual triage relative to the unfiltered initial anomaly set (58 raw flags reduced to 29 verified findings).

We caution, however, that an absolute FPR of 25.4% remains operationally significant in physical ICS environments: each false alarm can require engineering triage, temporary isolation of a PLC, restoration of a safe process state, and manual inspection of network and controller logs. MALF should therefore be deployed first in isolated testbeds or scheduled maintenance windows rather than directly within production control loops; in operational settings, FAA-flagged anomalies should be replayed against a digital twin or a spare controller before any production intervention is initiated. The benefit of FAA filtering is therefore comparative rather than absolute, and human-in-the-loop validation remains necessary in safety-critical deployments.

### 4.3. Comparative Performance Analysis

We first evaluate test case quality and diversity, then examine vulnerability discovery efficiency across heterogeneous industrial environments.

#### 4.3.1. Analysis of Test Case Quality and Diversity

**Figure 9:**
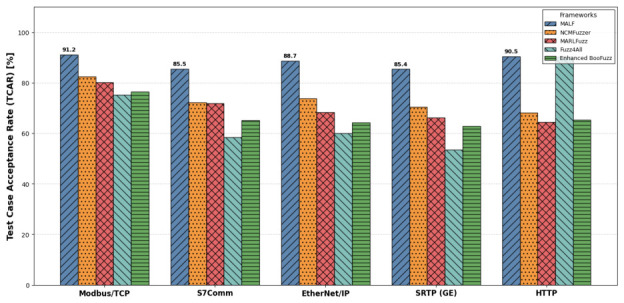
Test Case Acceptance Rate (TCAR) across protocols and frameworks.

Semantic Validity (TCAR Analysis): As summarized in Table 3 and Figure 9, MALF achieved an average TCAR of 88–92%, significantly outperforming Fuzz4All (≈75%) and Enhanced BooFuzz (≈71%). This gap highlights the critical role of the ISKG: while Fuzz4All leverages LLM generative power, it lacks domain constraints, frequently producing “hallucinated” packets rejected by PLC syntax parsers. The SGA’s ISKG-retrieved constraints enforce syntactic correctness before mutation, directing computational resources toward deep application-layer logic.

**Figure 10:**
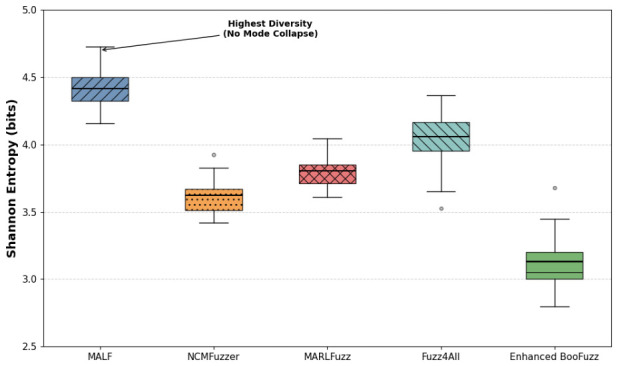
Mutation diversity of fuzzing frameworks (Shannon entropy).

Mutation Diversity (Shannon Entropy): As illustrated in Figure 10, MALF maintained the highest entropy levels (average 4.45 bits), markedly superior to MARLFuzz (≈3.8 bits, limited by RL mode collapse), NCMFuzzer (≈3.6 bits, constrained by heuristic rules), and Fuzz4All (≈4.0 bits, whose diversity stems from hallucinations rather than strategic variation). The QLoRA-tuned TGA introduces stochastic semantic variations while adhering to ISKG constraints, exploring a broader state space without degenerating into invalid noise.

**Figure 11:**
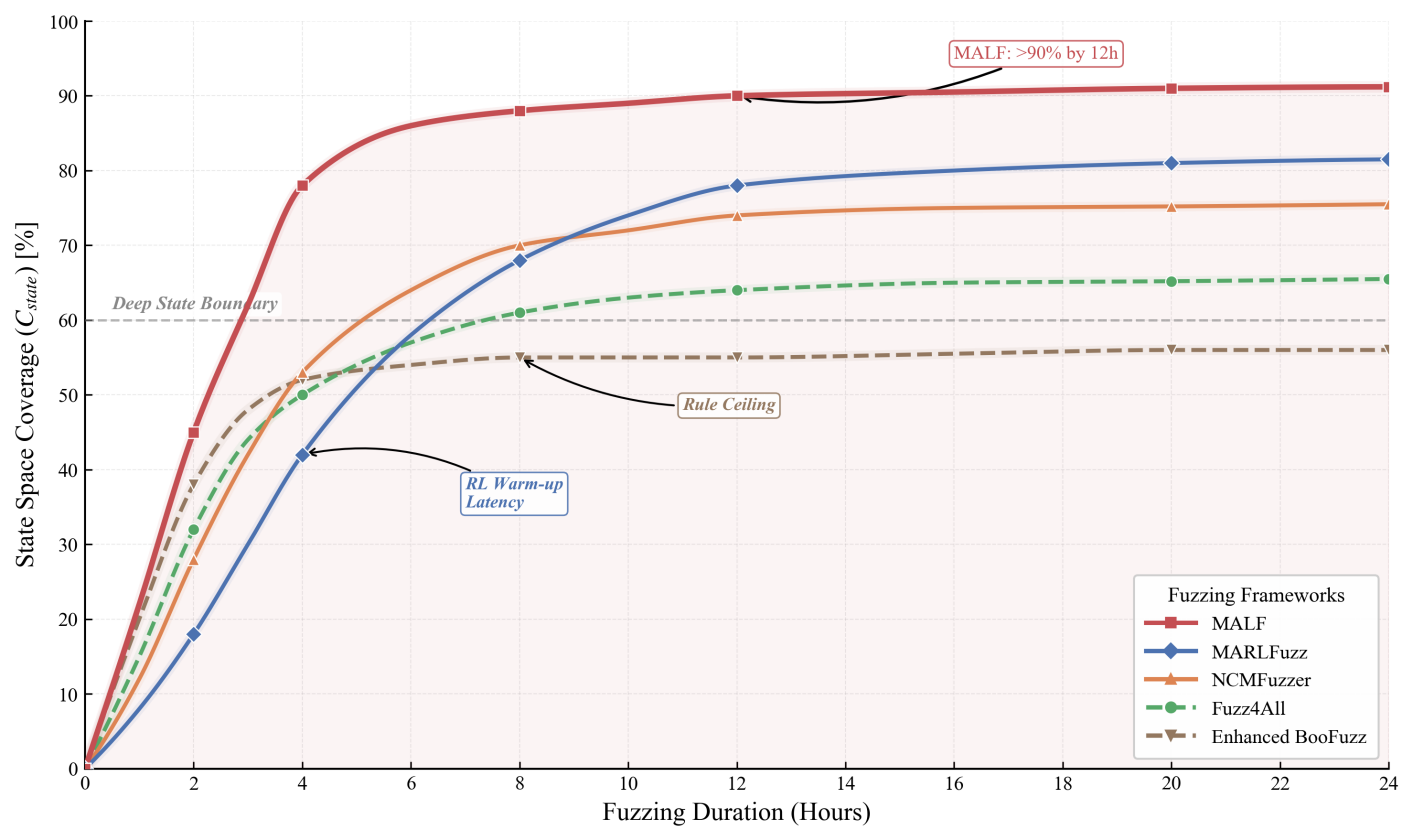
ISKG-defined state coverage ($C_{state}^{ISKG}$) across fuzzing frameworks. Higher values indicate broader traversal of states represented in the ISKG; the metric is graph-relative and does not measure firmware code coverage.

ISKG-Defined State Coverage ($C_{state}^{ISKG}$): As visualized in Figure 11, MALF achieved the highest ISKG-defined state coverage (>90%) within 12 h, reaching saturation (≈91%) by hour 16. BooFuzz plateaued at ≈56%, MARLFuzz converged to ≈81% after warm-up latency, and NCMFuzzer saturated at ≈75%. These results indicate that MALF more effectively traverses the ISKG-defined protocol states than the compared baselines. Because the state space is derived from the ISKG, this finding should be interpreted as evidence of stronger graph-relative exploration rather than as an ISKG-independent guarantee of firmware-level coverage; we discuss this limitation explicitly in Section 5.

**Figure 12:**
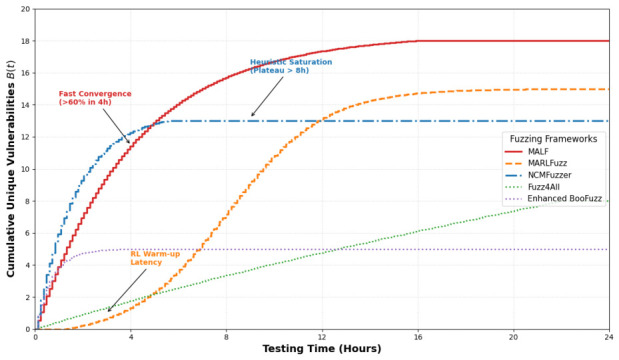
Cumulative vulnerability discovery B(t) over a 24-h fuzzing cycle.

**Figure 13:**
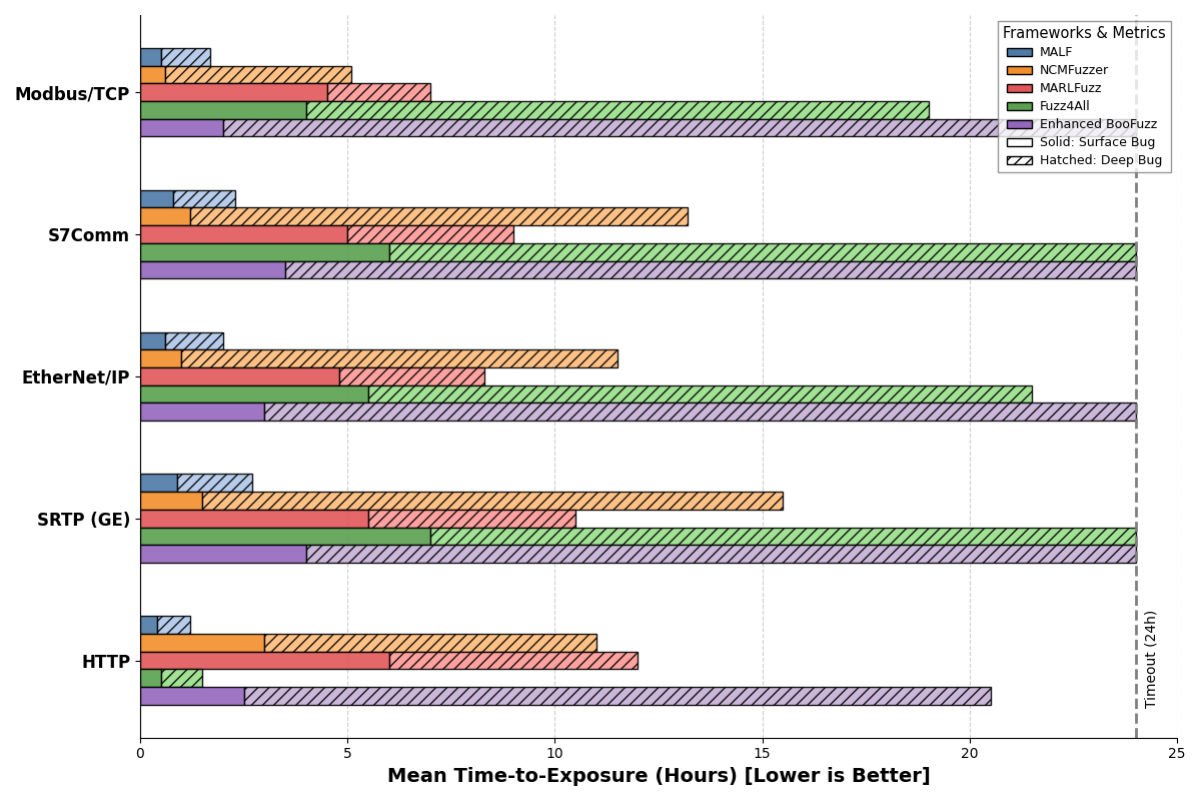
Time-to-Exposure (TTE) decomposed by vulnerability depth category. Shallow vulnerabilities require no prior protocol-state preparation; surface-state vulnerabilities require a syntactically valid session or service request; deep-state vulnerabilities require two or more ordered protocol transitions or vendor-specific state preconditions. Bars are means over N=5 independent 24 h runs; per-category sample sizes are shallow (n=12), surface-state (n=8), and deep-state (n=9).

Experimental Protocol and Statistical Significance: To account for the stochastic nature of fuzzing, each framework was evaluated over N=5 independent 24 h runs per protocol with different random seeds; the same seed schedule was applied across frameworks to control inter-run variance. All numerical values reported throughout Section 4.3—including those in Table 3 and Table 5, the ablation results in Table 6, and the curves in Figure 9, Figure 10, Figure 11, Figure 12 and Figure 13—are means over these five runs, and the inter-run standard deviation for TCAR, Shannon entropy (*H*), $C_{state}^{ISKG}$, N-Day Discovery Yield, and Time-to-Exposure remained below 4% of the corresponding mean for MALF on every protocol. Pairwise comparisons between MALF and each baseline were conducted using the Mann–Whitney U test (selected because the limited number of runs precludes a normality assumption), with Holm–Bonferroni correction applied uniformly across the metric × baseline grid; the corrected *p*-values were below 0.05 for $C_{state}^{ISKG}$, *H*, TCAR, and N-Day Discovery Yield against MARLFuzz, NCMFuzzer, and BooFuzz, and below 0.05 for $C_{state}^{ISKG}$ and TCAR against Fuzz4All. We caution that N=5 remains modest for stochastic fuzzing; the observed performance differences are therefore unlikely to be due to chance under the trials conducted, but large-scale repeated trials across additional deployments remain future work.

#### 4.3.2. Vulnerability Discovery Efficiency and Real-World Impact

We validated discovery capability using a benchmark of 15 known vulnerabilities (N-Day). As detailed in Table 4, MALF achieved a 100% detection rate, while baselines exhibited distinct blind spots: Fuzz4All failed against rigid binary protocols lacking ISKG-enforced structure; NCMFuzzer and MARLFuzz struggled with high-complexity logic flaws such as the Rockwell CIP Buffer Overflow (CVE-2020-12040). MALF’s ISKG-driven approach unifies detection across both shallow syntactic anomalies and deep-state semantic flaws, addressing RQ1.

Temporal Discovery Dynamics: Figure 12 plots the cumulative vulnerability discovery B(t) over a 24 h cycle. MALF exhibited a steep early-phase slope driven by ISKG-guided mutations, surpassing all baselines within the first 4 h and continuing to discover new vulnerabilities throughout the cycle. BooFuzz and NCMFuzzer plateaued early, while MARLFuzz displayed characteristic warm-up latency before stabilizing. Figure 13 decomposes the mean Time-to-Exposure (TTE) by vulnerability depth category, confirming that MALF’s advantage is the most pronounced for deep-state logic flaws. We define the three depth categories operationally as follows: a shallow vulnerability is triggered by a single malformed request issued in the initial or unauthenticated protocol state, requiring no prior session preparation; a surface-state vulnerability requires a syntactically valid session or service request but no multi-step state preparation; a deep-state vulnerability requires two or more ordered protocol transitions, session-resource manipulation, or vendor-specific state preconditions before the fault becomes reachable. This classification corresponds in spirit to the CVSS Attack Complexity dimension but is grounded in protocol-state preparation rather than environmental factors. Across the 29 unique vulnerabilities aggregated in Figure 13, the per-category sample sizes are shallow (n=12), surface-state (n=8), and deep-state (n=9).

Fuzzing Throughput and Discovery Yield: MALF’s average latency per test case is approximately 145 ms (ISKG retrieval ≈ 3 ms, QLoRA inference ≈ 125 ms, network execution ≈ 17 ms), sustaining ≈ 28 Execs/s with four parallel agents. Enhanced BooFuzz achieves ≈ 300 Execs/s, while Fuzz4All reaches ≈ 15 Execs/s due to multi-query auto-prompting.

Raw throughput is misleading when most test cases are parser-rejected. We define Effective Throughput $Execs_{eff}=Execs/s\times \bar{TCAR}$ and Discovery Yield $Y_{vuln}$ (unique vulnerabilities per $10^{5}$ effective executions). As shown in Table 5, MALF achieves a 20× higher N-Day Discovery Yield (0.703 vs. 0.035) and a 100% N-Day detection rate compared with BooFuzz’s 40%, confirming that ISKG-driven mutations are vastly more efficient at reaching deep-state vulnerabilities.

### 4.4. Ablation Study

**Figure 14:**
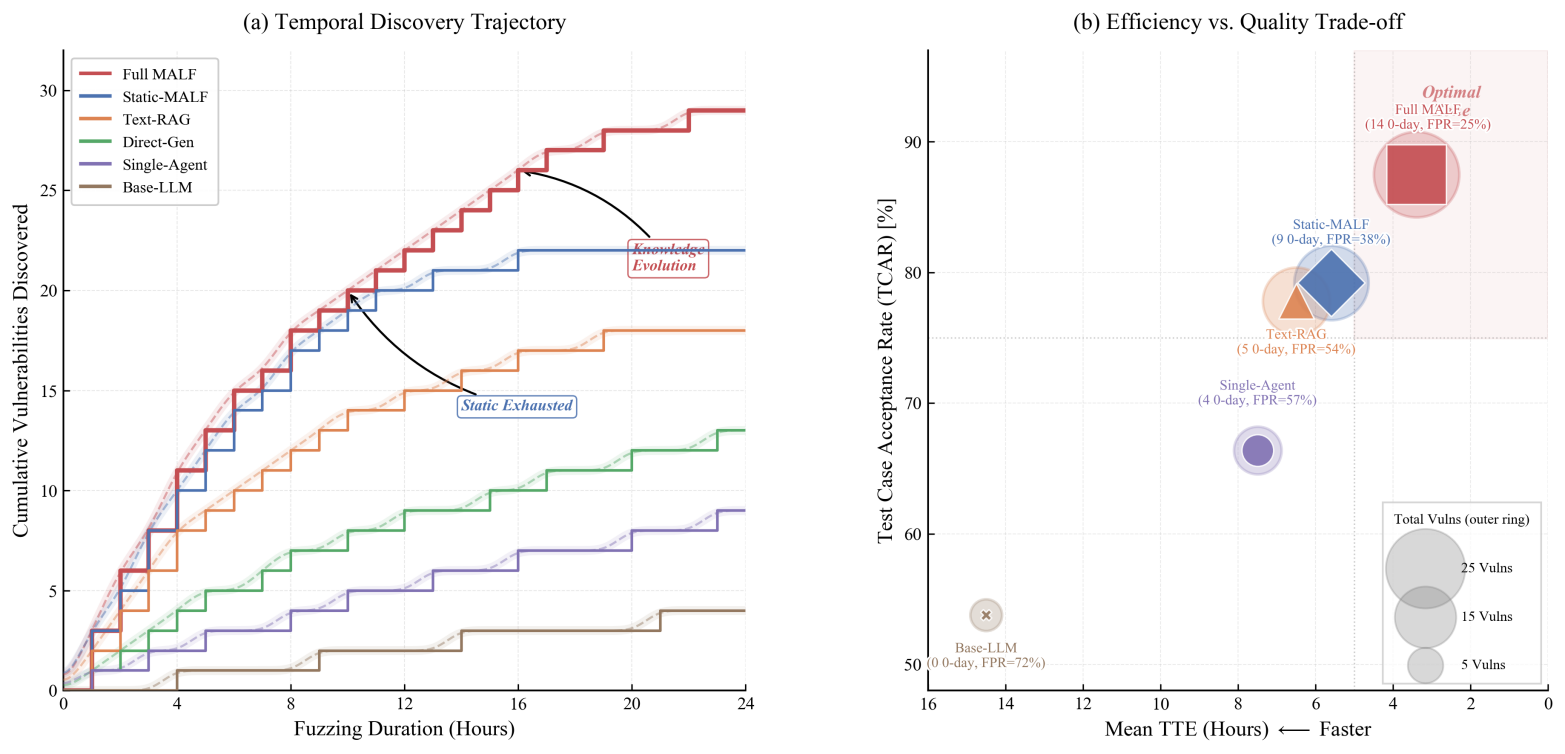
Performance comparison of MALF ablation variants across TCAR, FPR, and vulnerability discovery metrics.

To rigorously quantify the individual contributions of MALF’s core components—specifically the multi-agent architecture, CoT reasoning, Knowledge Evolution, and quantitative fine-tuning—we conducted a comprehensive ablation study. By systematically degrading specific modules while retaining others, we isolated the impact of each design decision on fuzzing efficiency and protocol compliance. The comparative results are summarized in Table 6 and Figure 14.

#### 4.4.1. Experimental Variants

Scope of the Ablation Configuration. For computational tractability, the ablation study was conducted on a controlled subset of the full evaluation configuration that comprises Modbus/TCP and S7Comm on Siemens S7-300 and Schneider TM221CE16T, together with EtherNet/IP on Rockwell 1769-L30ER. This subset was selected because it spans both standardized binary protocols and vendor-specific stateful behavior while keeping the cost of repeated degraded-variant testing manageable. Consequently, Table 6 should be interpreted as component-level evidence on this subset rather than as a full-protocol replacement for the comparative evaluation in Section 4.3; minor TCAR variance from Table 3 reflects both this scope difference and expected stochastic variation across independent trials.

We evaluated MALF against five degraded variants to isolate specific module effects:

Single-Agent (Monolithic): A unified agent performing seed generation, mutation, and analysis within a single context window. This variant assesses the value of architectural specialization.

Direct-Gen (w/o CoT): A multi-agent variant where the intermediate CoT reasoning steps are removed, forcing agents to directly generate payloads. This evaluates the role of structured reasoning.

Text-RAG MALF (w/o ISKG): The structured ISKG is replaced with a standard text-chunk RAG pipeline retrieving from the same vendor manuals via a vector database. All other components (multi-agent, CoT, QLoRA) remain identical. This isolates the specific value of structured graph-based knowledge over unstructured text retrieval.

Static-MALF (w/o Evolution): The ISKG is initialized with static specifications but remains frozen during testing. This isolates the contribution of the self-evolving knowledge loop.

Base-LLM (w/o QLoRA): Utilizing the base LLM without domain-specific fine-tuning, serving to verify the necessity of hexadecimal feature alignment.

#### 4.4.2. Impact of Architectural Collaboration and Reasoning

The ablation results in Table 6 validate agent specialization and explicit reasoning. The single-agent variant (TCAR 66.4%, FPR 57.3%, 9 vulns) suffers from context dilution—conflicting objectives within a single context window induce instruction drift. The Direct-Gen variant (TCAR 75.5%, FPR 47.1%) demonstrates that multi-agent coordination alone is insufficient; without CoT validation, the model hallucinated invalid field values and failed to systematically progress through protocol states.

#### 4.4.3. Impact of Knowledge Representation and Evolution

The Text-RAG variant (TCAR 77.8%, FPR 54.5%, 18 vulns with only five zero-days) isolates the value of structured graph knowledge versus unstructured text retrieval. The sharp FPR increase confirms that text-based RAG cannot capture multi-hop dependencies inherent in industrial state machines, establishing the ISKG as the primary driver of semantic precision.

The Base-LLM variant (TCAR 53.8%, FPR 72.5%, zero zero-days) confirms a critical modal gap: without QLoRA tuning, LLMs cannot align with hexadecimal constraints. Static-MALF (79.2% TCAR) suffers from premature saturation after 8 h, validating the self-evolving knowledge loop—Full MALF’s FPR improvement from 38.2% to 25.4% demonstrates progressive refinement of the FAA’s discrimination capability (answering RQ2).

### 4.5. Case Studies

Table 7 summarizes all 14 previously unknown findings across eight vendors. Of these, four have been formally assigned CNVD identifiers (CNVD-2024-16009, CNVD-2025-22875, CNVD-2025-29811, and CNVD-2026-06041), while the remaining 10 candidates are still under vendor review at the time of writing. Below, we detail the exploitation logic for three representative CNVD-assigned cases.

#### 4.5.1. Case Study 1: Stateful Logic Exhaustion on Rockwell 1769-L30ER (CNVD-2024-16009)

Phase 1: Knowledge Retrieval. Upon target identification, the SGA queried the ISKG and retrieved a critical constraint regarding the CIP Connection Manager: “The device maintains a finite session pool, requiring strict Forward_Open/Forward_Close handshakes.” This highlighted a potential Unreleased Resource Pattern.

Phase 2: Agent Orchestration. Guided by this pattern, the TGA formulated a stateful exhaustion strategy. Instead of malformed packets, it generated a stream of valid Forward_Open(0x54) requests while intentionally suppressing the mandatory Forward_Close (0x4E) messages. The agent’s CoT reasoning aimed to “orphan connection states” to force the PLC to hold resources indefinitely.

**Figure 15:**
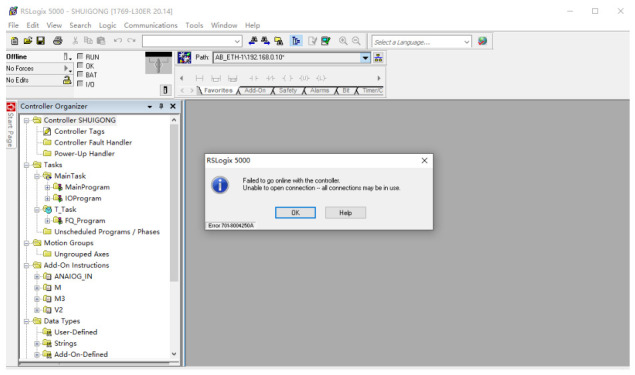
“Connection Timed Out” error on Studio 5000.

Phase 3: Physical Consequence. Within 15 s, the PLC’s session table saturated, rejecting all legitimate connections. As shown in Figure 15, the engineering workstation reported a “Connection Timed Out,” confirming a successful Denial of Service (DoS) unique to stateful protocol abuse.

#### 4.5.2. Case Study 2: Sequence-Dependent Buffer Overflow on GE VersaMax (CNVD-2025-22875)

Phase 1: Knowledge Retrieval. The ISKG identified Port 18245 as a legacy interface requiring a “Two-Stage Interaction.” Retrieved specifications indicated that a preamble packet is necessary to initialize the command buffer before execution instructions can be processed.

Phase 2: Agent Orchestration. Exploiting this dependency, the TGA constructed a multi-packet sequence: (1) a 56-byte “Null Packet” to reset the parser; (2) a malformed command packet (Opcode 02 00 50) injected with repetitive 00 a0 padding. The reasoning engine inferred that Command 0x50 triggers a memory copy, where missing delimiters in the second stage would likely cause a stack overrun.

**Figure 16:**
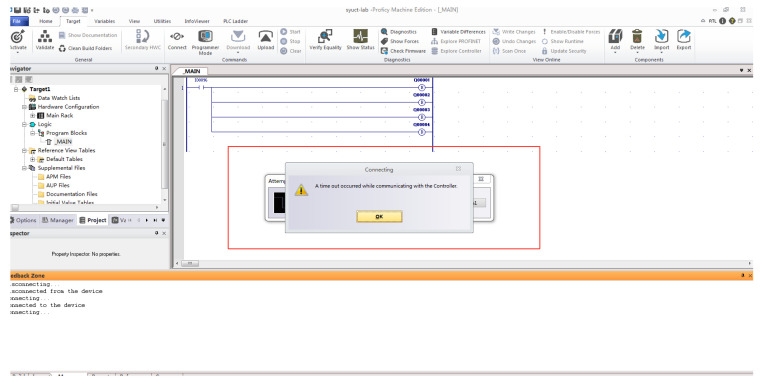
“Target No Response” error on GE VersaMax.

Phase 3: Physical Consequence. The impact was immediate. The PLC’s network stack crashed completely, ceasing to respond to ICMP or TCP requests. Figure 16 illustrates the “Target No Response” state, requiring a physical power cycle to restore functionality.

#### 4.5.3. Case Study 3: Malformed Header Processing on Inovance EZ320 (CNVD-2025-29811)

Phase 1: Knowledge Retrieval. For this closed-source protocol, MALF leveraged Dynamic Behavior Mining. The SGA analyzed traffic traces to infer a fixed header signature (0x13 00) followed by variable parameters, dynamically updating the ISKG.

Phase 2: Agent Orchestration. The TGA adopted a “Hollow Payload” strategy: maintaining the valid header to bypass initial checks, but nullifying the parameter block after the two-byte instruction marker 0x47 0x01 (the hexadecimal sequence 47 01, identified during traffic mining as the proprietary opcode that initiates a parameter-write transaction on the EZ320). The instruction marker 47 01 alone is not the complete payload; it precedes a variable-length parameter block, which the agent intentionally replaced with null bytes so that the parser would attempt to process an undefined parameter set, triggering unhandled exceptions.

**Figure 17:**
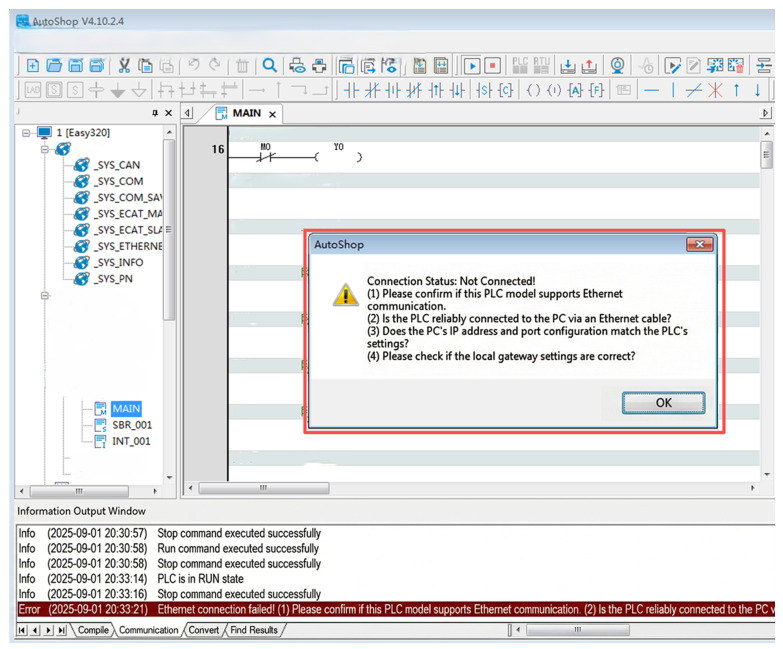
“Failing to discover the device” error on AutoShop.

Phase 3: Physical Consequence. Upon processing this sequence, the PLC entered a “Zombie State”—the physical run light remained active, but the EtherNet port became unresponsive. Figure 17 shows the failure of vendor software to discover the device, proving MALF’s capability to compromise undocumented proprietary protocols.

These case studies across heterogeneous vendors—from standard EtherNet/IP to undocumented proprietary protocols—provide empirical evidence for RQ3, confirming MALF’s generalization capability across diverse IIoT environments.

### 4.6. Responsible Disclosure

All discovered vulnerabilities were reported to the respective vendors or to CNVD following coordinated disclosure procedures. Four vulnerabilities have received confirmed CNVD identifiers (CNVD-2024-16009, CNVD-2025-22875, CNVD-2025-29811, and CNVD-2026-06041). The remaining 10 discoveries are under vendor review. To facilitate reproducibility, Proof-of-Concept (PoC) payloads for the 15 N-Day benchmark vulnerabilities are publicly available at https://github.com/Mewtwoz/n-days-poc-benchmark-and-dataset (accessed on 20 March 2026).

## 5. Discussion

The empirical results validate the central hypothesis: structured knowledge grounding transforms LLM-based fuzzing from a generative novelty into a reliable security testing methodology for IIoT.

Knowledge Representation as the Critical Factor. The ablation study reveals that the choice of knowledge representation—not merely the presence of domain knowledge—determines testing quality. The Text-RAG variant, accessing identical information through unstructured retrieval, exhibited a 54.5% FPR compared to Full MALF’s 25.4%. Industrial protocol constraints require explicit relational encoding (graph triples) rather than approximate semantic similarity matching. This finding has broader implications for deploying LLM agents in safety-critical domains where structured reasoning is essential.

Efficiency–Trustworthiness Synergy. MALF’s lower raw throughput (28 Execs/s vs. BooFuzz’s 300 Execs/s) is more than offset by semantic precision: on the N-Day benchmark, MALF attains a 20× higher Discovery Yield than BooFuzz (0.703 vs. 0.035 unique findings per $10^{5}$ effective executions). This validates the design principle that semantic depth outweighs brute-force volume in IIoT fuzzing, where protocol state machines are deep but narrow. The deterministic crash-verification mechanism further ensures that flagged anomalies reflect genuine vulnerabilities rather than transient network artifacts, a capability absent in all evaluated baselines.

Limitations. Three constraints bound the interpretation of our results. First, $C_{state}^{ISKG}$ is graph-relative: states absent from the ISKG are also absent from the denominator, so reported coverage is conditional on graph completeness, and the complementary $C_{resp}$ (Equation (17)) only partially mitigates this circularity. Second, although the ISKG ingests solely public specifications and passive baseline PCAPs, those PCAPs originate from the same laboratory deployment as the evaluation devices; the reported performance therefore reflects a testbed-informed grey-box scenario rather than a fully device-independent guarantee, and cross-vendor zero-shot deployment remains future work. Third, the 25.4% post-verification FPR, while substantially below the unfiltered anomaly rate, is still operationally significant in safety-critical ICS settings and should be paired with digital-twin or spare-controller validation before any production intervention. We further note that ISKG construction still requires 24–48 h of expert-assisted setup per protocol and that Shannon entropy captures byte-level rather than semantic diversity—an effect partially offset by the simultaneous achievement of high entropy and high TCAR.

## 6. Conclusions

This paper presented MALF, a Knowledge-Driven Multi-Agent LLM Fuzzing Framework for automating vulnerability discovery across heterogeneous IIoT protocols. By coupling an Industrial Security Knowledge Graph (ISKG) with QLoRA-tuned generative agents and a self-evolving knowledge loop, the framework supports effective, efficient, and trustworthy security testing in proprietary industrial environments.

On a testbed spanning ten vendors and five protocols, MALF achieves an average TCAR of 88.3% and 91.2% ISKG-defined state coverage. On the 15-vulnerability N-Day benchmark, MALF reaches 100% detection with an N-Day Discovery Yield of 0.703 unique findings per $10^{5}$ effective executions—a 20× improvement over BooFuzz (0.035) and a 5× improvement over MARLFuzz—while reducing the post-verification False Positive Rate to 25.4%, down from 54.5% under an ablation that replaces the ISKG with text-chunk retrieval. In a separate real-world campaign, MALF further identifies 14 previously unknown vulnerability candidates, of which four have been assigned CNVD identifiers (CNVD-2024-16009, CNVD-2025-22875, CNVD-2025-29811, CNVD-2026-06041) and 10 remain under vendor review. These results provide controlled-testbed evidence that knowledge-grounded AI agents can systematically expose deep-state logic flaws that evade conventional methods.

We caution that the reported numbers correspond to a testbed-informed grey-box scenario: the ISKG ingests only public specifications and passive baseline PCAPs, but those PCAPs were collected from the same laboratory deployment as the evaluation devices, and the ISKG-defined coverage is graph-relative rather than equivalent to firmware code coverage. The 25.4% False Positive Rate, while a substantial reduction, also remains operationally significant in safety-critical ICS settings and should not be interpreted as fit for direct deployment in production control loops without digital-twin or spare-controller validation. Future work will focus on (i) automating ISKG construction from protocol specifications and traffic traces to reduce expert effort; (ii) extending evaluation to cross-vendor zero-shot scenarios in production-representative deployments; and (iii) scaling multi-agent coordination to heterogeneous, large-scale industrial networks, with the aim of advancing trustworthy AI-driven security practices for critical IIoT infrastructure.

## Figures and Tables

**Table 1:** FAA anomaly scoring and mutation-modulation parameters used in the reported experiments.

Parameter	Value	Rationale
$w_{1}$	0.50	Weight assigned to error-category severity ($E_{type}$); dominant term to prioritize fault-class signals over timing or resource fluctuations.
$w_{2}$	0.30	Weight assigned to latency deviation ($T_{latency}$) from the protocol-specific baseline derived from passive PCAPs.
$w_{3}$	0.20	Weight assigned to resource-consumption indicators ($R_{resource}$) such as connection-pool exhaustion or CPU-cycle anomalies.
$\tau_{critical}$	0.70	Threshold above which deterministic replay-based crash verification is triggered.
β	0.50	Scaling factor governing the transition from broad exploration to focused exploitation in Equation (10).

**Table 2:** Details of the device under test.

Manuf.	PLC Model	Monitor	Supported Protos
SIEMENS AG, Munich, Germany	S7-300	STEP 7 (V5.6)	S7Comm, UDP
	S7-1200	TIA Portal (V15.1)	HTTP, S7Comm
	S7-416 SoftPLC	TIA Portal (V15.1)	Profinet, S7Comm
Rockwell Automation Inc., Milwaukee, WI, USA	1766-L32BWA	RSLogix 500 (V12.01)	ENIP, SNMP
	1769-L30ER	Studio 5000 (V32.01)	ENIP, TCP/IP
	1756-L81E	Studio 5000 (V32.01)	ENIP, TCP/IP
	1789-L60 SoftLogix	Studio 5000 (V32.01)	ENIP
Mitsubishi Electric Corporation, Tokyo, Japan	FX5U-32MR/ES	GX Works3 (V1.060N)	SLMP, Melsoft
	FX5U-32MT/ES	GX Works3 (V1.060N)	SLMP, Melsoft
Emerson Electric Co. St. Louis, USA	VersaMax IC200 V9.5	PAC Machine (V9.5)	SRTP, SNP
OMRON Corporation, Kyoto, Japan	CP2E-N14RDR-A	CX-Program (V9.73)	FINS (TCP/UDP)
Panasonic Industry Co., Tokyo, Japan	SFDOC32ET	FPWIN GR7 (Ver.2.2)	MEWTOCOL
Schneider Electric SE, Rueil-Malmaison, France	TM221CE16T	EcoStruxure (V1.1)	Modbus/TCP
Phoenix Contact GmbH & Co. KG, Blomberg, Germany	MAX100-24T	PC Worx (6.0)	Profinet, Modbus
Delta Electronics Inc., Taipei, China	AS228T-A	ISPSoft (V3.11)	EtherNet/IP
Shenzhen Inovance Technology Co., Shenzhen, China	EZ320-0808TP	AutoShop (V4.10)	Modbus/TCP

**Table 3:** Quantitative analysis of test case quality and diversity across protocols.

Protocol	MALF			MARLFuzz			NCMFuzzer			Fuzz4All			BooFuzz		
	**TCAR**	H	$C_{\mathrm{st}}$	**TCAR**	H	$C_{\mathrm{st}}$	**TCAR**	H	$C_{\mathrm{st}}$	**TCAR**	H	$C_{\mathrm{st}}$	**TCAR**	H	$C_{\mathrm{st}}$
Modbus	91.2	4.52	92.5	73.8	3.92	80.1	78.5	3.65	74.2	72.1	4.10	65.3	71.5	2.85	56.0
S7Comm	86.5	4.38	90.8	68.2	3.75	78.5	72.1	3.52	72.0	65.3	3.95	62.8	65.0	2.70	52.5
EIP	88.3	4.45	91.2	70.5	3.82	79.2	74.8	3.60	73.5	68.7	4.02	64.0	67.2	2.78	54.2
SRTP	87.8	4.40	89.5	69.0	3.70	77.8	70.2	3.48	71.0	63.5	3.88	61.5	64.8	2.65	51.8
HTTP	87.5	4.48	92.0	69.5	3.85	81.5	71.5	3.55	75.8	68.0	4.05	68.5	65.5	2.80	55.0
**Avg.**	**88.3**	**4.45**	**91.2**	70.2	3.81	79.4	73.4	3.56	73.3	67.5	4.00	64.4	66.8	2.76	53.9

**Table 4:** Lateral comparison of fuzzing tools on known vulnerabilities (N-Day benchmark).

Manufacturer	Model	Protocol	Vulnerability Description	Ref (CVE/CNVD)	MALF	NCM	MARL	F4A	Boo
Siemens	S7-300	Profinet	Remote Denial of Service	CVE-2019-13946	•	•	•	∘	•
	S7-300	S7 Comm	Port 102 Input Validation Failure	CVE-2015-2177	•	•	•	∘	•
	S7-300	Profibus	I/O Module Stoppage (DoS)	CVE-2015-0015	•	∘	∘	∘	∘
	S7-1200	HTTP	HTTP Response Splitting (CRLF)	CVE-2014-2909	•	∘	∘	•	∘
	S7-1200	HTTP	Cross-Site Scripting (XSS)	CVE-2012-3040	•	∘	∘	•	∘
	S7-1200	ISO-TSAP	Crafted Packet Crash (DoS)	CVE-2013-0700	•	•	•	∘	•
	S7-1200	S7 Comm	Diagnostic Buffer Info Disclosure	CVE-2012-3037	•	•	∘	∘	∘
Rockwell	1766-L32	EIP (PCCC)	Stack Buffer Overflow	CNVD-2018-00883	•	•	∘	∘	∘
	1766-L32	EIP	EtherNet Interface DoS	CVE-2016-5645	•	•	•	∘	•
	SoftLogix	EIP (CIP)	Buffer Overflow in CIP Stack	CVE-2020-12040	•	∘	∘	∘	∘
Emerson	VersaMax	SRTP	Memory Corruption (RCE)	CNVD-2013-13377	•	•	•	∘	∘
Omron	CP2E	FINS	CPU Cycle Time Error (DoS)	CVE-2015-0987	•	•	•	∘	•
Schneider	TM221	Modbus	CPU Crash via Func 0x71	CVE-2015-7937	•	•	•	∘	•
	TM221	HTTP	Web Service DoS via POST	CVE-2018-7789	•	∘	∘	•	∘
CODESYS	Linux SL	OPC UA	OPC UA Protocol Stack DoS	CVE-2021-29241	•	•	∘	•	∘
**Detection Rate Summary**					**100%**	**60%**	**47%**	**27%**	**40%**

*Note: • detected; ∘ missed*.

**Table 5:** Fuzzing throughput and Discovery Yield analysis.

Framework	Raw (Ex/s)	$\bar{\mathrm{TCAR}}$	${\mathrm{Execs}}_{\mathrm{eff}}$/s	Vulns	$Y_{\mathrm{vuln}}$
BooFuzz	**300**	66.8%	**200.4**	6	0.035
Fuzz4All	15	67.5%	10.1	4	0.460
NCMFuzzer	120	73.4%	88.1	9	0.118
MARLFuzz	45	70.2%	31.6	7	0.256
**MALF**	28	**88.3%**	24.7	**15**	**1.362**

$Y_{vuln}$: Unique N-Day vulnerabilities per $10^{5}$ effective executions over 24 h. Zero-day discoveries from the real-world testbed are reported separately in Section 4.5; they are excluded from this benchmark to preserve a like-for-like comparison.

**Table 6:** Performance of MALF ablation variants on the controlled subset configuration (Modbus/TCP and S7Comm on Siemens S7-300 and Schneider TM221CE16T; EtherNet/IP on Rockwell 1769-L30ER). Metrics are computed on this subset and are not directly comparable to the five-protocol numbers in Table 3.

Variant	Agents	Reas.	Knowledge	TCAR	FPR	Vulns (0-Day)	TTE
Full MALF	Multi	CoT	Dynamic	87.5%	25.4%	29 (14)	3.4
Static	Multi	CoT	Static	79.2%	38.2%	22 (9)	5.6
Text-RAG	Multi	CoT	Text DB	77.8%	54.5%	18 (5)	6.5
Direct-Gen	Multi	None	Dynamic	75.5%	47.1%	13 (5)	N/A ^†^
Single	Mono	CoT	Dynamic	66.4%	57.3%	9 (4)	7.5
Base-LLM	Multi	CoT	Dynamic	53.8%	72.5%	4 (0)	14.5

Vulns: Total (N-Day + 0-Day). Values in parentheses denote confirmed 0-Days. ^†^ **N/A**: The Direct-Gen variant relies on sporadic, unguided mutations, requires manual adjustment and cannot systematically progress through deep protocol states, rendering a meaningful mean TTE incalculable within the 24 h cycle.

**Table 7:** Summary of zero-day vulnerabilities and critical anomalies discovered by MALF in real-world PLCs.

Vendor & Model	Protocol	Vulnerability Class	Root Cause & Impact	Status / ID
***Siemens***				
S7-300	Profibus	Denial of Service	I/O module crash via abnormal traffic sequence	Pending
S7-1200	S7 Comm	Denial of Service	Stack design defect handling malformed packets	Pending
***Rockwell Automation (Allen-Bradley)***				
**1769-L30ER**	**EtherNet/IP**	**Resource Exhaustion**	**Connection hang prevents new sessions**	**CNVD-2024-16009**
1766-L32BWA	SNMP	Denial of Service	SNMP set processing error sequence	Pending
1766-L32BWA	EtherNet/IP (CIP)	Logic Error	NVRAM mismatch via abnormal write	Pending
***Emerson (GE)***				
VersaMax IC200	SRTP / SNP	Memory Corruption	Buffer overflow in legacy parsing stack	**CNVD-2026-06041**
**VersaMax IC200**	**SRTP / SNP**	**Denial of Service**	**Malformed packet sequence crashes network stack**	**CNVD-2025-22875**
***Mitsubishi***				
FX5U-32MR	SLMP	Logic Error	CPU memory clear via specific instruction	Pending
FX5U-32MR	Melsoft UDP	Denial of Service	Inherited protocol stack defect from FX3U	Pending
***Omron***				
CP2E-N14RDR	FINS	Denial of Service	Malformed `Write Parameter’ operation	Pending
***Panasonic***				
SFDOC32ET	MEWTOCOL	Abnormal Execution	Program loss via specific instruction sequence	Pending
***Others***				
Delta AS228T	EtherNet/IP	Denial of Service	Packet processing error on Port 44818	Pending
**Inovance EZ320**	**TCP**	**Buffer Overflow**	**Service port DoS via malformed payloads**	**CNVD-2025-29811**
Inovance EZ320	TCP	Buffer Overflow	Specific overflow on Port 12939	Pending

*Rows in bold indicate the specific case studies detailed in Section 4.5*.

## Data Availability

Proof-of-Concept (PoC) payloads for the 15 N-Day benchmark vulnerabilities and the QLoRA training dataset description are publicly available at https://github.com/Mewtwoz/n-days-poc-benchmark-and-dataset (accessed on 20 March 2026). Due to the sensitive nature of zero-day vulnerability details, full exploit payloads will be released following completion of the coordinated disclosure process with affected vendors.

## DURC Statement

Current research is limited to the field of IIoT cybersecurity, which is beneficial for improving the security posture of critical infrastructure and does not pose a threat to public health or national security. Authors acknowledge the dual-use potential of the research involving vulnerability discovery tools and confirm that all necessary precautions have been taken to prevent potential misuse. All discovered vulnerabilities were reported to affected vendors through coordinated disclosure via CNVD before publication. As an ethical responsibility, authors strictly adhere to relevant national and international laws about DURC. Authors advocate for responsible deployment, ethical considerations, regulatory compliance, and transparent reporting to mitigate misuse risks and foster beneficial outcomes.
